# C5‐C5aR1‐mediated immune responses during fungal infection: Clinical and translational implications

**DOI:** 10.1002/ctm2.1424

**Published:** 2023-09-18

**Authors:** Jigar V. Desai, Michail S. Lionakis

**Affiliations:** ^1^ Fungal Pathogenesis Section, Laboratory of Clinical Immunology and Microbiology (LCIM) National Institute of Allergy and Infectious Diseases (NIAID) National Institutes of Health (NIH) Bethesda Maryland USA; ^2^ Present address: Center for Discovery and Innovation, Hackensack Meridian Health, Nutley, NJ, USA

Systemic fungal infections continue to pose a substantial health challenge worldwide, resulting in considerable morbidity and mortality. These infections primarily affect immunocompromised patient populations, which are continuously expanding, in part due to the increasing use of immune pathway‐targeting biologics for the treatment of neoplastic and autoimmune disorders.^1^ Available antifungal treatments are limited in their in vivo effectiveness, often accompanied by significant side effects. Hence, there is an unmet need to better understand the mechanisms by which our immune system combats such infections, as this could lead to the development of personalized risk stratification, prognostication, vaccination and therapeutic strategies and improve patient outcomes.^2^

As a pivotal component of innate immunity, the complement system comprises a complex network of effector proteins that contribute to opsonization, inflammation and bacterial lysis, providing a crucial line of defense against bacterial pathogens.^3^ Indeed, patients with inherited deficiency or pharmacological blockade of C5 are at‐risk for developing life‐threatening systemic infections by encapsulated bacteria such as meningococcus and pneumococcus.^4^, ^5^ We recently examined the role of the complement C5a‐C5aR1 axis in the context of systemic fungal infections.^6^ This line of research emerged from the unexpected clinical finding of opportunistic fungal infections ‐such as systemic candidiasis and aspergillosis‐ developing in patients receiving the Food and Drug Administration (FDA)‐approved, C5‐targeting monoclonal antibody, eculizumab,^5^, ^7^ which highlighted the unanticipated critical role of C5a‐C5aR1 in antifungal host defense.

In this work, we used several orthogonal in vitro, murine and human studies and analyses to demonstrate the pivotal role of the anaphylatoxin and chemoattractant C5a in licensing myeloid phagocytes to exert sterilizing immunity during systemic candidiasis via engaging its cognate receptor C5aR1.^6^ By employing a well‐established murine model of systemic candidiasis in which myeloid phagocyte‐dependent responses are critical for effective host defense and the kidney is the primary target organ,^2^, ^8^ we found an essential role for C5a‐C5aR1 in promoting tissue fungal clearance and host survival, whereas C5aR2, the other C5a cognate receptor, was dispensable.^6^ Mechanistically, C5a‐C5aR1 was redundant for the recruitment of neutrophils and monocytes to the infected tissue. By contrast, C5a‐C5aR1 was critical for the accumulation of renal macrophages after infection by promoting their Extracellular signal‐regulated kinase (ERK)‐ and Protein kinase B (AKT)‐mediated survival through C5a‐driven inhibition of caspase‐dependent apoptosis and pyroptosis (Figure 1).^6^ This finding expands upon previous reports that have implicated chemoattractant receptor signaling in promoting myeloid cell survival.^9^, ^10^ Moreover, C5a‐C5aR1 was crucial for orchestrating certain antifungal effector functions of myeloid phagocytes in the infected kidney. Specifically, C5a‐C5aR1 mediated fungal uptake by neutrophils ‐via enhancing their expression of bona fide phagocytic receptors such as Dectin‐1 and FcγR1‐ and promoted non‐oxidative fungal killing by macrophages (Figure 1), whereas neutrophil extracellular trap formation, neutrophil fungal killing and macrophage fungal uptake were intact in the setting of C5aR1 deficiency.^6^

**FIGURE 1:**
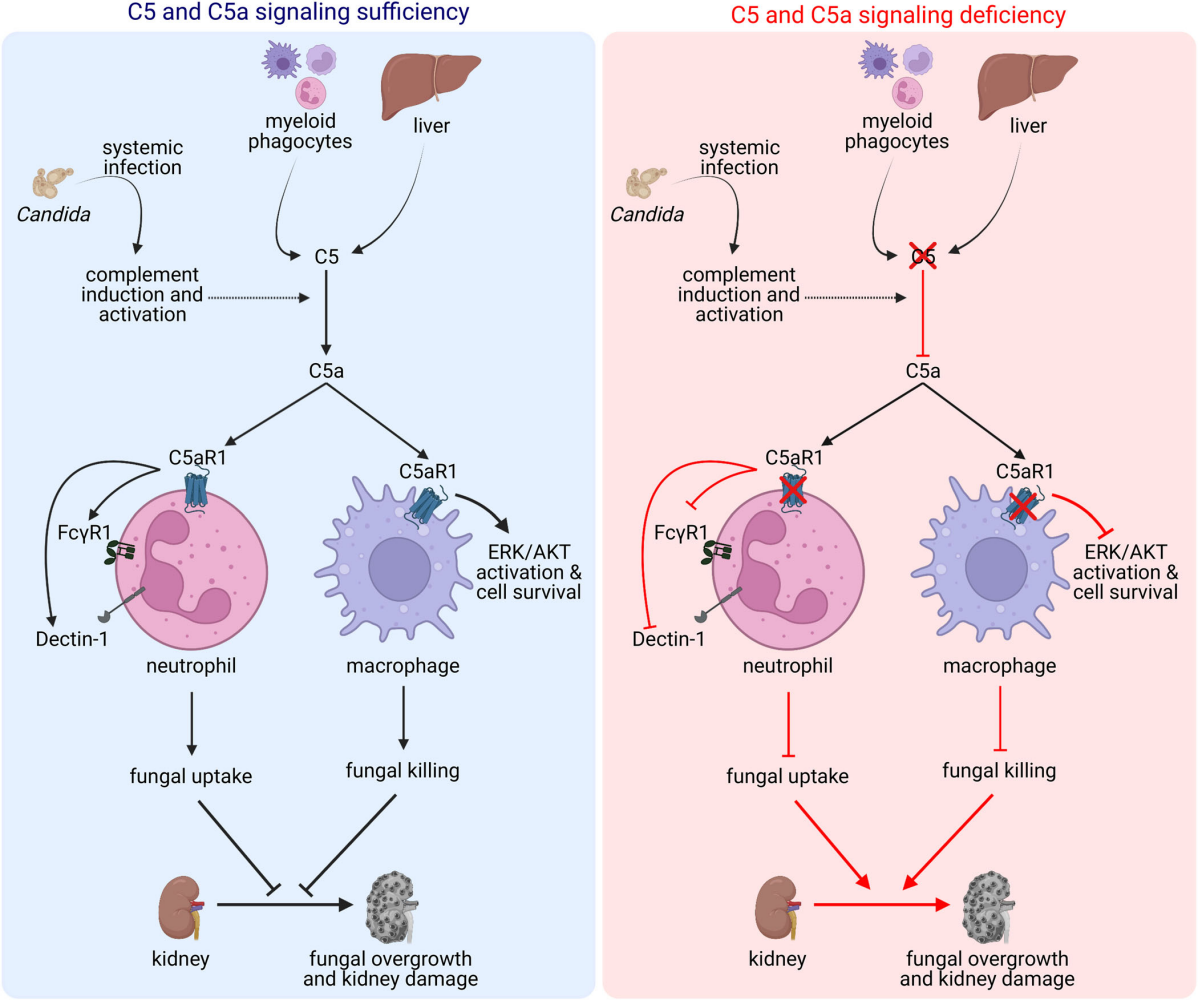
C5a‐C5aR1‐driven sterilizing immunity against systemic candidiasis. In the setting of systemic candidiasis, C5 is produced by hepatocytes and intrinsically by myeloid phagocytes locally at the site of renal infection and is converted to C5a during complement activation. C5a binds to its cognate receptor, C5aR1, to promote the antifungal effector function of myeloid phagocytes enabling tissue fungal clearance and host survival. Engagement of C5aR1 on neutrophils promotes the uptake of *Candida* through the induction of bona fide phagocytic receptors such as Dectin‐1 and FcγR1. Engagement of C5aR1 on macrophages mediates the non‐oxidative killing of *Candida* and promotes their survival and accumulation in the infected tissue by providing Extracellular signal‐regulated kinase (ERK)‐ and Protein kinase B (AKT)‐dependent survival signals that inhibit caspase‐dependent apoptosis and pyroptosis. In C5aR1 deficiency, neutrophil fungal uptake as well as macrophage survival, tissue accumulation, and fungal killing are impaired, collectively resulting in inexorable tissue fungal proliferation, renal injury, and host mortality.

Two additional notable mechanistic insights arose from this work. First, C5aR1 deficiency resulted in the metabolic rewiring of renal macrophages, which exhibited increased glycolysis and enhanced mTOR signaling in vivo. Pharmacological targeting of increased mTOR signaling with the FDA‐approved drug, rapamycin, ameliorated the macrophage survival defect, improved renal function and increased host survival following fungal infection in the setting of C5aR1 deficiency.^6^ Second, beyond the well‐recognized role of hepatocyte‐derived C5 in promoting antimicrobial activity, our study unveiled the critical contribution of C5 that is produced locally in the infected kidney by renal myeloid phagocytes in antifungal host defense, as mice lacking C5 specifically in myeloid phagocytes exhibited increased tissue fungal burden and decreased survival after *Candida* infection.^6^ This finding represents the first direct in vivo demonstration of the involvement of extrahepatic, cell‐intrinsic, intracellular complement production in antimicrobial host defense and expands on the previously reported functions of intracellular complement production in the settings of sterile inflammation and infection‐related immunopathology.^11^, ^12^, ^13^ Additional studies are required to further define the roles of intracellular complement production, termed ‘the complosome’, and its molecular regulation in the context of other infections and non‐infectious inflammatory conditions.

Furthermore, to establish the potential clinical relevance of these discoveries, the study employed several experimental approaches to translate the mouse findings into the human context. First, we evaluated the FDA‐approved C5aR1 inhibitor, avacopan, and showed that avacopan‐exposed human neutrophils and monocyte‐derived macrophages exhibited the same functional antifungal defects that were observed in C5aR1‐deficient mouse phagocytes.^6^ This parallel in functionality strengthens the link between mouse and human immune responses in the setting of C5aR1 deficiency and suggests that avacopan may heighten the risk for systemic fungal infections in patients similarly to eculizumab. Second, the study identified a complement transcriptional module that was markedly induced in candidemic patients and was highly predictive for candidemia in hospitalized patients.^6^ This finding deepens our understanding of the human innate immune response to this infection and shows promise for the eventual development of personalized immune‐based tools to complement existing diagnostic platforms in candidemia. Third, the study demonstrated an independent correlation between suboptimal serum C5a levels at the time of diagnosis of candidemia and decreased survival of candidemic patients.^6^ This finding further underscores the clinical relevance of the complement system's role in combating fungal infections and emphasizes the potential importance of measuring C5a levels as an individualized immune‐based prognostication strategy in candidemic patients. Fourth, the study identified a significant independent correlation between a single nucleotide polymorphism (SNP) in the *C5* gene that is associated with decreased *C5* mRNA levels in human blood leukocytes and poor clinical outcomes in candidemic patients, namely persistent fungemia.^6^ This genetic variation further highlights the complex interplay between host genetics and susceptibility to fungal infections^14^, ^15^, ^16^, ^17^ and uncovers this *C5* SNP as a potential factor for personalized risk assessment and treatment in candidemic patients.

In summary, these research findings have important implications for fungal infection pathogenesis, diagnosis and treatment. They shed light on the vital role of the C5a‐C5aR1 axis in activating phagocytes, which drives sterilizing immunity against systemic fungal infections. The human findings of the induced complement transcriptional module, C5a serum levels at diagnosis of infection and the *C5* SNP collectively provide a foundation for devising improved personalized risk stratification, prognostication and therapeutic strategies in candidemic patients. Moreover, the introduction in clinical practice of other biologics targeting complement effector proteins such as C3, which was also shown to be critical for innate antifungal responses during systemic candidiasis in this study,^6^ suggests that these biologics may also increase the risk of life‐threatening fungal infections in vulnerable patients, thereby warranting increased clinical awareness, vigilance and surveillance.

## CONFLICT OF INTEREST STATEMENT

The authors declare that no conflict of interest exists.
